# Correction: Latent tuberculosis infection in foreign-born communities: Import vs. transmission in The Netherlands derived through mathematical modelling

**DOI:** 10.1371/journal.pone.0198376

**Published:** 2018-05-24

**Authors:** Hester Korthals Altes, Serieke Kloet, Frank Cobelens, Martin Bootsma

The graph B2 in S2 Fig is unreadable due to formatting errors. The graphs C3 and C4 are missing from S2 Fig. Please view the correct S2 Fig below.

## Supporting information

S2 Fig Tornadoplot for estimated transmission parameter (1), percentage contribution to LTBI from TB transmission within the Netherlands (2), from immigration (3), or from travel to country of origin (4) for Moroccan (A), Turkish (B) and Indonesians (C). Percentage deviation from the estimate in main text (Table 2) is given for the minimum and maximum values for the parameters in Table 1 in the main text.
(PDF)
