# Supplementary material for: Latent tuberculosis infection in foreign-born communities: Import vs. transmission in The Netherlands derived through mathematical modelling
Source: PLoS One. 2018 Feb 14;13(2):e0192282. doi: 10.1371/journal.pone.0192282 (PMC5812587; doi:10.1371/journal.pone.0192282)
Supplement: S1 Table — (PDF) [file pone.0192282.s001.pdf]

S1 Table: First- and second-generation TB cases in the Netherlands 1993-2013

Country of origin	1st generation immigrants	2nd generation, parents same country of origin	2nd generation, parents different countries of origin	Percentage all 2nd generation/total immigrants	Percentage 2nd generation non-mixed parents/total immigrants
**Turkey**	1186	45	67	8.6%	3.7%
**Morocco**	2515	133	118	9.1%	5.0%
**Indonesia**	992	50	127	15.1%	4.8%
