# Supplementary material for: Latent tuberculosis infection in foreign-born communities: Import vs. transmission in The Netherlands derived through mathematical modelling
Source: PLoS One. 2018 Feb 14;13(2):e0192282. doi: 10.1371/journal.pone.0192282 (PMC5812587; doi:10.1371/journal.pone.0192282)
Supplement: S2 Table — (PDF) [file pone.0192282.s002.pdf]

S2 Table: Prevalence all forms of TB in country of origin

Year	Turkey*	Morocco*	Indonesia#
**1995**	58	219	483
**1996**	56	208	481
**1997**	56	195	479
**1998**	49	180	478
**1999**	47	164	476
**2000**	45	152	474
**2001**	42	143	453
**2002**	38	136	432
**2003**	34	132	411
**2004**	30	129	390
**2005**	28	128	369
**2006**	26	126	356
**2007**	25	126	344
**2008**	25	127	331
**2009**	25	128	319
**2010**	24	129	306
**2011**	24	131	301
**2012**	23	133	297
**2013**	23	134	293

* obtained from (1)
#obtained from (2); highlighted values are linearly extrapolated

References

1. WHO TB burden estimates [Internet]. 2014 [cited 30-03-2015]. Available from: http://www.who.int/tb/country/data/download/en/.
2. World Health Organization (WHO). Global Tuberculosis Report. 2013.
