# Supplementary material for: Latent tuberculosis infection in foreign-born communities: Import vs. transmission in The Netherlands derived through mathematical modelling
Source: PLoS One. 2018 Feb 14;13(2):e0192282. doi: 10.1371/journal.pone.0192282 (PMC5812587; doi:10.1371/journal.pone.0192282)
Supplement: S3 Table — (PDF) [file pone.0192282.s003.pdf]

S3 Table: Immigration and emigration from Morocco, Turkey and Indonesia 1995-2013

Year	Morocco*		Turkey*		Indonesia¶	
	Immigration	Emigration	Immigration	Emigration	Immigration	Emigration
**1995**	3193	2217	4717	3013	1228	1158
**1996**	4528	2490	6209	4189	1362	1225
**1997**	4894	1711	6291	2637	1357	1011
**1998**	5535	1466	5460	2210	1858	929
**1999**	4670	1432	4817	1860	1749	870
**2000**	4482	1105	5393	1158	1931	852
**2001**	5235	1103	5993	1286	2021	793
**2002**	5192	1396	6181	1601	1969	1133
**2003**	4894	1567	6703	2148	1755	1217
**2004**	3655	1605	4580	2831	1527	1191
**2005**	2356	1981	3393	2872	1370	1322
**2006**	2085	2392	3175	3321	1432	1449
**2007**	1724	2249	2855	2879	1548	1375
**2008**	2117	2065	4048	2728	1589	1348
**2009**	2388	1496	4099	2522	1433	1290
**2010**	2371	1541	4460	3099	1560	1230
**2011**	2675	1546	4065	3321	1693	1311
**2012**	2272	1807	3887	4028	1468	1312
**2013**	2326	1512	3809	4447	1605	1248

*Statline 02.2015 (1)
¶Statline 07.2016 (1)

Reference

1. Immi- en emigratie naar geboorteland, leeftijd (31 december) en geslacht [Internet]. 2016 [cited 02.2015 (Turkey, Morocco); 07.2016 (Indonesia)]. Available from: http://statline.cbs.nl/Statweb/publication/?DM=SLNL&PA=03742&D1=0-1&D2=0&D3=0&D4=92,135,236&D5=0&D6=a&HDR=G4,G1,G2,T,G3&STB=G5&VW=T.
