# Supplementary material for: Latent tuberculosis infection in foreign-born communities: Import vs. transmission in The Netherlands derived through mathematical modelling
Source: PLoS One. 2018 Feb 14;13(2):e0192282. doi: 10.1371/journal.pone.0192282 (PMC5812587; doi:10.1371/journal.pone.0192282)
Supplement: S4 Table — (PDF) [file pone.0192282.s004.pdf]

S4 Table: Immigration by model compartment

	Morocco					Turkey					Indonesia				
**Year**	S	E_1	E_2	I_1	I_2	S	E_1	E_2	I_1	I_2	S	E_1	E_2	I_1	I_2
**1995**	2395	79	712	6	1	4250	74	670	6	0	921	30	270	7	0
**1996**	3396	112	1009	9	2	5594	99	882	9	1	1022	34	303	2	2
**1997**	3671	121	1093	8	1	5668	100	898	5	1	1018	34	303	3	0
**1998**	4152	137	1235	8	3	4919	86	775	8	2	1394	46	414	4	0
**1999**	3503	116	1046	3	2	4340	76	683	7	3	1312	43	386	5	3
**2000**	3361	111	998	10	2	4859	86	769	5	1	1448	47	425	11	0
**2001**	3926	130	1169	7	3	5399	95	859	3	1	1516	50	448	6	1
**2002**	3894	129	1160	9	0	5569	99	885	4	0	1477	48	435	8	1
**2003**	3670	121	1087	13	3	6039	107	953	8	0	1316	43	387	7	2
**2004**	2741	91	818	4	1	4126	73	652	5	1	1145	37	335	8	1
**2005**	1767	59	528	1	1	3057	55	488	1	0	1028	34	302	6	1
**2006**	1564	52	466	2	1	2860	50	454	2	0	1074	35	317	3	3
**2007**	1293	43	383	5	0	2572	45	409	2	0	1161	38	343	5	1
**2008**	1587	53	474	1	2	3647	65	578	3	0	1192	39	351	7	0
**2009**	1790	60	536	1	1	3693	66	589	0	0	1075	36	320	3	0
**2010**	1778	59	531	2	1	4018	71	640	2	0	1170	38	344	7	1
**2011**	2006	67	601	1	0	3662	65	585	0	0	1270	42	377	4	0
**2012**	1704	56	508	3	1	3502	63	560	0	0	1101	36	326	3	2
**2013**	1745	58	522	1	0	3432	61	549	0	0	1204	40	358	3	0
