# Supplementary material for: Latent tuberculosis infection in foreign-born communities: Import vs. transmission in The Netherlands derived through mathematical modelling
Source: PLoS One. 2018 Feb 14;13(2):e0192282. doi: 10.1371/journal.pone.0192282 (PMC5812587; doi:10.1371/journal.pone.0192282)
Supplement: S1 Appendix — (PDF) [file pone.0192282.s005.pdf]

S1 Appendix. Mathematical formulation of system of ordinary equations, describing fluxes from the susceptible, latently infected and diseased compartments

Fig A: Schematic of fluxes in system of ordinary differential equations

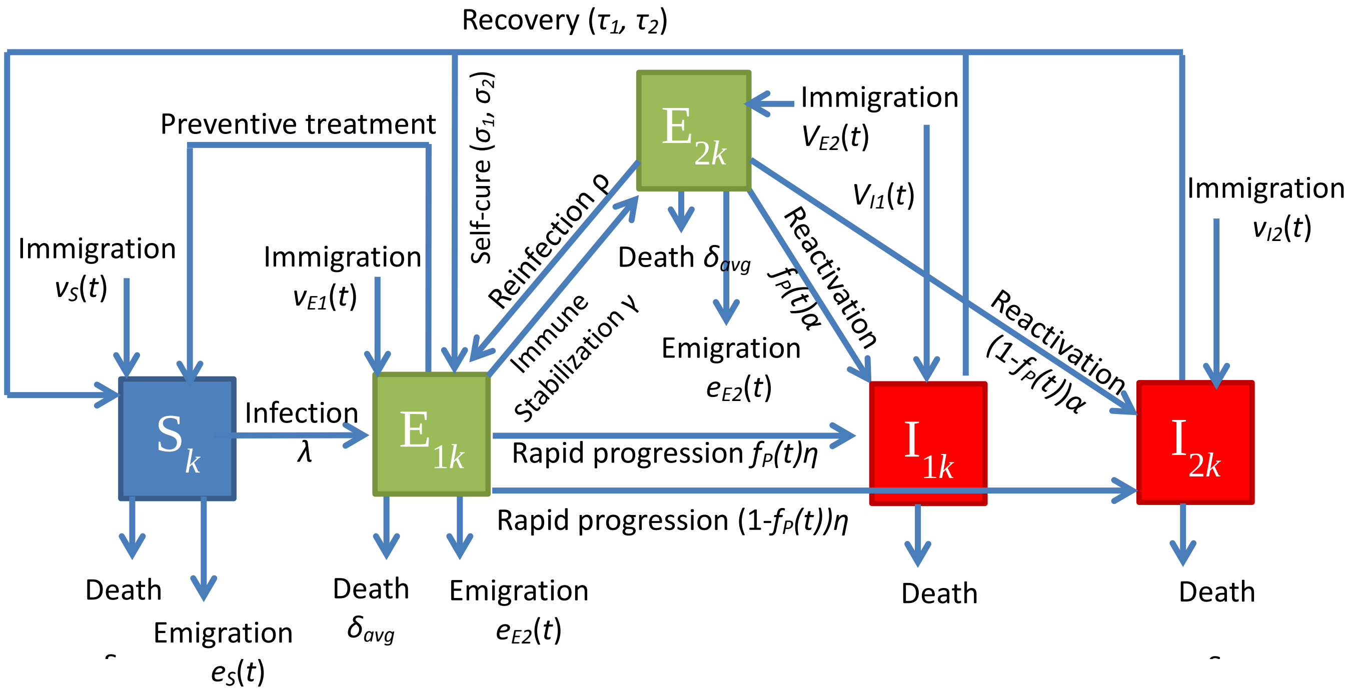

$$
\begin{cases}
\frac{dS}{dt} = v_S(t) - w(t)\left(\frac{S(t)}{S(t)+E_1(t)+E_2(t)}\right) - \lambda(t)S(t) - \delta_{avg}S(t) + \kappa E_1(t) + \tau_1 I_1(t) + \tau_2 I_2(t) \\
\frac{dE_1}{dt} = v_{E1}(t) - w(t)\left(\frac{E_1(t)}{S(t)+E_1(t)+E_2(t)}\right) + c\lambda(t)E_2(t) + \lambda(t)S(t) - (\eta + \gamma + \kappa + \delta_{avg})E_1(t) + \sigma_1 I_1(t) + \sigma_2 I_2(t) \\
\frac{dE_2}{dt} = v_{E2}(t) - w(t)\left(\frac{E_2(t)}{S(t)+E_1(t)+E_2(t)}\right) - (c\lambda(t) + \alpha + \delta_{avg})E_2(t) + \gamma E_1(t) \\
\frac{dI_1}{dt} = v_{I1}(t) + f_P(t)[\alpha E_2(t) + \eta E_1(t)] - (\delta_1 + \tau_1 + \sigma_1)I_1(t) \\
\frac{dI_2}{dt} = v_{I2}(t) + (1 - f_P(t))[\alpha E_2(t) + \eta E_1(t)] - (\delta_2 + \tau_2 + \sigma_2)I_2(t)
\end{cases}
$$

Where:

$$\lambda(t) = \psi\,(1-\nu)\frac{I_1(t)}{S(t)+E_1(t)+E_2(t)+I_1(t)+I_2(t)} + \psi\,\nu\,\pi(t)$$

$$f_P(t) = f_{P0} - bt$$

where f_{P0} is the fraction of the average number of yearly PTB or EPTB cases over the 1994-1995.

$w(t)$ is the yearly number of emigrants for each ethnic group, as given in S3 Table.

Global parameters, *i.e.* not country-dependent, are indicated in red. Country-specific parameters are indicated in green. All time-dependent variables differ by country.

Start values

The population sizes of the different ethnic groups are calculated based on the numbers from CBS (1). So the total first generation immigrants from country k, N_k, has to be calculated as it is only directly available from CBS starting in 1996:

$N_k(1995) = N_k(1996) + emigration_k(1995) - immigration_k(1995)$.

If we assume that the number of (pulmonary and extrapulmonary) TB cases does not change rapidly over the years (roughly steady state value), we can calculate the number of (pulmonary and extrapulmonary) TB in 1995 as follows:

$I_{1\_k}(1995)$=yearly average number of new PTB cases in 1994-1995 / $(\tau_1+\sigma_1+\delta_1)$

$I_{2\_k}(1995)$= yearly average number of new EPTB cases in 1994-1995 / $(\tau_2+\sigma_2+\delta_2)$

$E_{1\_k}(1995)=N_k(1995)*f_{IGRA\_k}*r_{E1\_k}$

$E_{2\_k}(1995)= N_k(1995)*f_{IGRA\_k}*(1-r_{E1\_k})$

Where f_{IGRA_k} is the proportion of immigrants entering the Netherlands from country k, having a positive IGRA (Interferon Gamma Release Assay) test - as a proxy for LTBI (2):

$f_{IGRA\_Morocco}=0.24$; $f_{IGRA\_Indonesia}=0.25$; $f_{IGRA\_Turkey}=0.16$

r_{E1_k} is the fraction of LTBI attributable to recent infection in 1995 and is estimated through model fitting.

And where:

$N_{Morocco}(1995)=139596$

N_{Turkey}(1995)=165544

$N_{Indonesia}$(1995)=148890

$f_{P0\text{-}Morocco}$=99/(99+66.5);

$f_{P0\text{-}Turkey}$=55/(27+55);

$f_{P0\text{-}Indonesia}$=(99+440)/(453+99+440); For Indonesians, the model with $f_P(t) = f_{P0}$ gave a better fit than the model with a non-zero slope $f_P(t) = f_{P0}\text{-}bt$.

We therefore considered $f_{P0\text{-} Indonesia}$ the fraction of PTB over all TB cases over the entire study period rather than only 1994-1995.

References

1. Bevolking, huishoudens en bevolkingsontwikkeling; vanaf 1899 [Internet]. 2014 [cited 4-02-2015]. Available from: http://statline.cbs.nl/Statweb/publication/?DM=SLNL&PA=37556&D1=61,76,139,145-146,149,155-156&D2=94-113&HDR=T&STB=G1&VW=T.
2. Mulder C, van Deutekom H, Huisman EM, Toumanian S, Koster BF, Meijer-Veldman W, et al. Role of the QuantiFERON(R)-TB Gold In-Tube assay in screening new immigrants for tuberculosis infection. Eur Respir J. 2012;40(6):1443-9.
