# Supplementary material for: Latent tuberculosis infection in foreign-born communities: Import vs. transmission in The Netherlands derived through mathematical modelling
Source: PLoS One. 2018 Feb 14;13(2):e0192282. doi: 10.1371/journal.pone.0192282 (PMC5812587; doi:10.1371/journal.pone.0192282)
Supplement: S2 Appendix — (PDF) [file pone.0192282.s006.pdf]

S2 Appendix. Travel rate to country of origin

The 'travel term' consists of the TB transmission parameter ψ_k , the travel rate v_k and the prevalence in country of origin $\pi_k(t)$. The travel rate was derived as follows for the Moroccans:

Moroccan TB cases: 9% stayed more than 3 months, 47% 1-3 months, 25% less than 1 month;

Moroccan controls: 1% stayed more than 3 months, 36% 1-3 months, 21% less than 1 month (1). The average length of stay was set to 4 months, 2 months and 0.5 month respectively.

The fraction of the time immigrants resided in Morocco then becomes:

v_M=[0.09*$fraction_{MoTBNL}$+0.01*(1- $fraction_{MoTBNL}$)]*4/12+[0.47* $fraction_{MoTBNL}$ +0.36*(1- $fraction_{MoTBNL}$)]*2/12+[0.25* $fraction_{MoTBNL}$ +0.21*(1- $fraction_{MoTBNL}$)]*0.5/12=0.07

where $fraction_{MoTBNL=}$0.00067/4 is TB prevalence Moroccans in the Netherlands (assuming an average duration of TB disease of 3 months to compute prevalence based on incidence numbers).

This yields v_M=0.072.

For the Turkish community we computed:

$fraction_{TuTBNL}$=0.0003/4

v_T =(0.13* $fraction_{TuTBNL}$ +0.03*(1- $fraction_{TuTBNL}$))*4/12+(0.31* $fraction_{TuTBNL}$ +0.33*(1- $fraction_{TuTBNL}$))*2/12+(0.13* $fraction_{TuTBNL}$ +0.23*(1- $fraction_{TuTBNL}$))*0.5/12

and v_T =0.075.

As we found no literature on traveling frequency in Indonesians, we took v_I =0.02 as an educated guess: about 3 times lower than Turkish and Moroccan travelling rates as Indonesia is much further away.

Reference

1. Kik SV, Mensen M, Beltman M, Gijsberts M, van Ameijden EJ, Cobelens FG, et al. Risk of travelling to the country of origin for tuberculosis among immigrants living in a low-incidence country. Int J Tuberc Lung Dis. 2011;15(1):38-43.
