# Supplementary material for: Latent tuberculosis infection in foreign-born communities: Import vs. transmission in The Netherlands derived through mathematical modelling
Source: PLoS One. 2018 Feb 14;13(2):e0192282. doi: 10.1371/journal.pone.0192282 (PMC5812587; doi:10.1371/journal.pone.0192282)
Supplement: S3 Appendix — (PDF) [file pone.0192282.s007.pdf]

S3 Appendix. Maximum likelihood fitting procedure

We applied a maximum likelihood (ML) method to find the best fit for our target parameters b, ψ and r_{E1}. Since LTBI data records are incomplete -the majority are undetected- and the NTR data file contains no information about susceptibles, we use the data about PTB and EPTB separately. The model is fitted to two lists of observations t_1, t_2 ….. t_n, where t_i are the dates (in days) that the NTR registered the event 'individual diagnosed with PTB' and 'individual diagnosed with EPTB'. Second, we identify the part of our model that corresponds to these observations. The number of PTB and EPTB cases diagnosed per year is $\tau_1 I_1$ and $\tau_2 I_2$, respectively. We assume that the events 'individual diagnosed with (E)PTB' are Poisson distributed.

The likelihood of observing the PTB cases is $L_P(b,\psi, r_{E1})$ can be written as follows:

$$L_P(b,\psi,r_{E1}) = e^{-\int_0^T \mu_P(b,\psi,r_{E1},t).dt}.\prod_{i=1}^{n_P} \mu_P(b,\psi,r_{E1},t_i)$$

T is the end of the observation period, n is the number of observations, and $\mu_P(b,\psi, r_{E1},t)$ is the function describing the rate of the event 'PTB case diagnosed'. Therefore $\mu_P(b,\psi, r_{E1},t) = \tau_1. I_1(b,\psi, r_{E1},t)$.

Roughly speaking, $L_P(b,\psi,r_{E1})$ describes the hazard rate of an event at t_i, $1\leq i \leq n_P$ (the second term), multiplied by the probability that no event took place before, after or in between (the first term).

We can write the same equation for EPTB notifications, where $\mu_E(\mathrm{b},\psi, r_{E1},t) = \tau_2. I_1(b,\psi, r_{E1},t)$ is the function describing the rate of the event 'EPTB case diagnosed':

$$L_E(b,\psi,r_{E1}) = e^{-\int_0^T \mu_E(b,\psi,r_{E1},t).dt}.\prod_{i=1}^{n_E} \mu_E(b,\psi,r_{E1},t_i)$$

The overall likelihood is the product of the two likelihoods:

$$L(b,\psi,r_{E1}) = L_P(b,\psi,r_{E1}).L_E(b,\psi,r_{E1})$$

If we take its logarithm, it is the sum of the two log-likelihoods:

$$l(b,\psi, r_{E1})=\log(L) = -\int_0^T \mu_P(b,\psi,r_{E1},t).dt + \sum_{i=1}^{n_P} \log \mu_P(b,\psi,r_{E1},t_i) - \int_0^T \mu_E(b,\psi,r_{E1},t).dt + \sum_{i=1}^{n_E} \log \mu_E(b,\psi,r_{E1},t_i)$$

Maximizing this function will give the maximum likelihood estimates for the increase in EPTB detection over time, TB transmission rate, and fraction recent LTBI in 1995.
