# Supplementary material for: Latent tuberculosis infection in foreign-born communities: Import vs. transmission in The Netherlands derived through mathematical modelling
Source: PLoS One. 2018 Feb 14;13(2):e0192282. doi: 10.1371/journal.pone.0192282 (PMC5812587; doi:10.1371/journal.pone.0192282)
Supplement: S4 Appendix — (PDF) [file pone.0192282.s008.pdf]

S4 Appendix. Smear-positive versus smear-negative cases

As studies have reported a much lower infectiousness of smear-negative PTB cases compared to smear-positive PTB cases (1, 2), we have considered the scenario in which infectious TB cases are the smear-positive PTB, and the non-infectious TB cases are EPTB and the smear-negative PTB.

As smear status was unknown in 15% of Moroccan, Turkish and Indonesian PTB cases in the Netherlands over the period 1995-2013, it was imputed as follows. Within each ethnic group, smear-positivity was assigned to cases with a missing smear-status, by binomially drawing from a distribution with probability p_{sp} of being smear-positive in a particular year (as measured in the cases with known status).

Table A gives the estimated parameters (a.o. transmission rates), as well as the percentage contribution to LTBI by different routes; the best-fit values and the yearly smear-positive PTB cases / non-infectious TB are shown in Fig. A. When only considering the smear-positive PTB as infectious, we find a higher transmission rate for all ethnic groups than when all PTB are considered infectious (see transmission rates in Table A and in Table 2, main paper). This difference stems from the fact that fewer cases transmit infection in this scenario, with relatively more secondary cases. The other parameters derived are in line with estimates in the main paper (except slope of proportion developing smear-positive PTB).

Contribution to LTBI by different is hardly affected, the greatest difference being for transmission in NL for Moroccans, rising to 46% (Table A) from 44% (Table 2).

Table A: Immigration by model compartment

	Transmission parameter smear-positive PTB 1995-2013	Fraction recent LTBI (1995)	Slope of proportion developing smear-positive PTB	Contribution to LTBI* 1995-2013 %		
**Country of birth**	/year	%	/year	Transmission in NL	Immigration	Travel country origin
Morocco	48.05	12	-0.02	46.15	43.78	10.08
Turkey	25.43	9	-0.03	25.75	70.62	3.63
Indonesia	24.79	2	-	27.19	61.98	10.83

*Taking into account the contribution of self-cured into the recent LTBI

Fig A: Yearly number of smear-positive PTB cases, and smear-negative PTB and EPTB ("non-infectious TB") respectively in Moroccan (A-B), Turkish (C-D), and Indonesian cases (E-F). Bars represent number of cases (registered and imputed), the line represents the best fit curve.

(A)

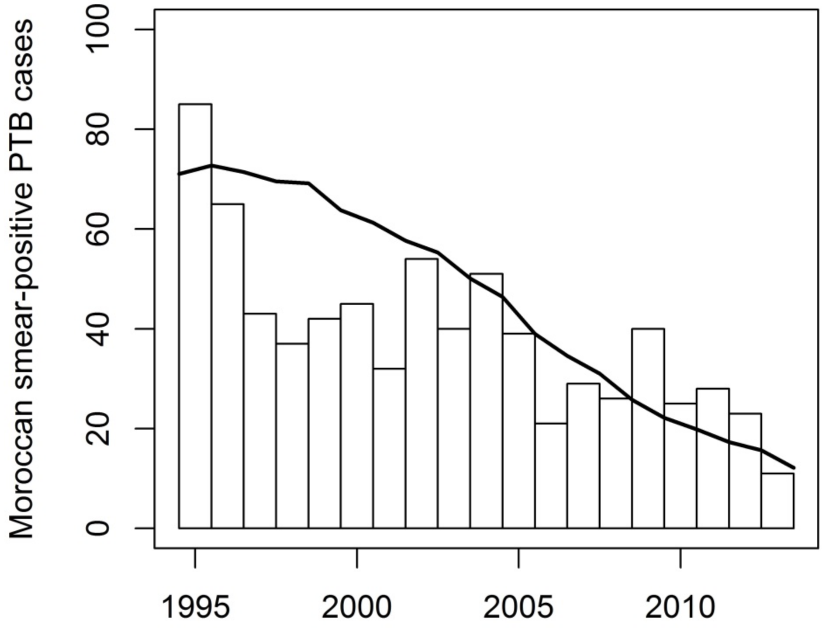

(B)

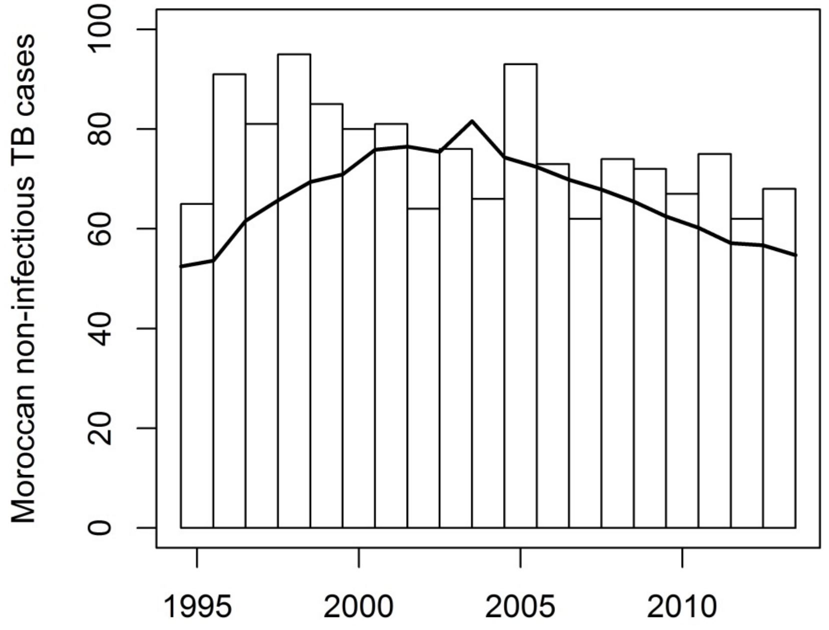

(C)

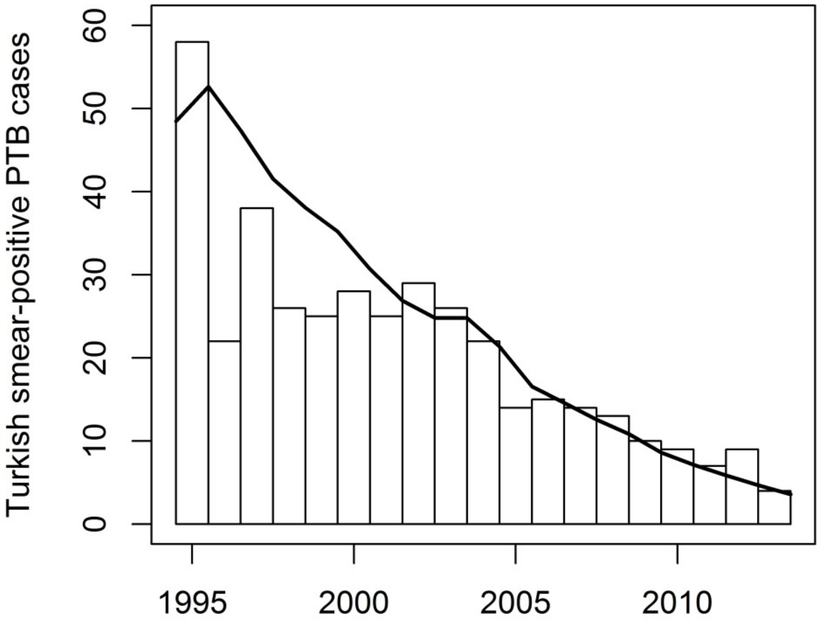

Turkish smear-positive PTB cases
0
10
20
30
40
50
60
1995
2000
2005
2010

(D)

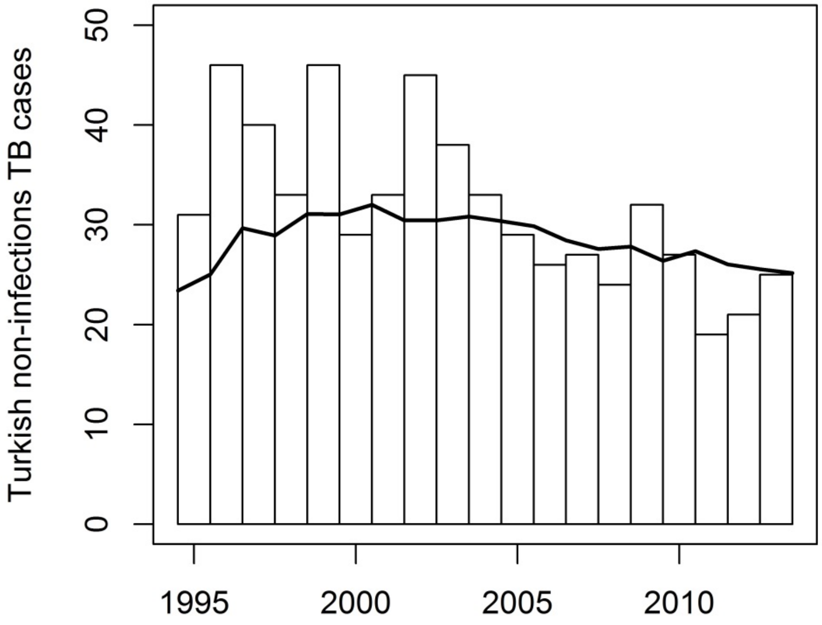

Turkish non-infections TB cases
0
10
20
30
40
50
1995
2000
2005
2010

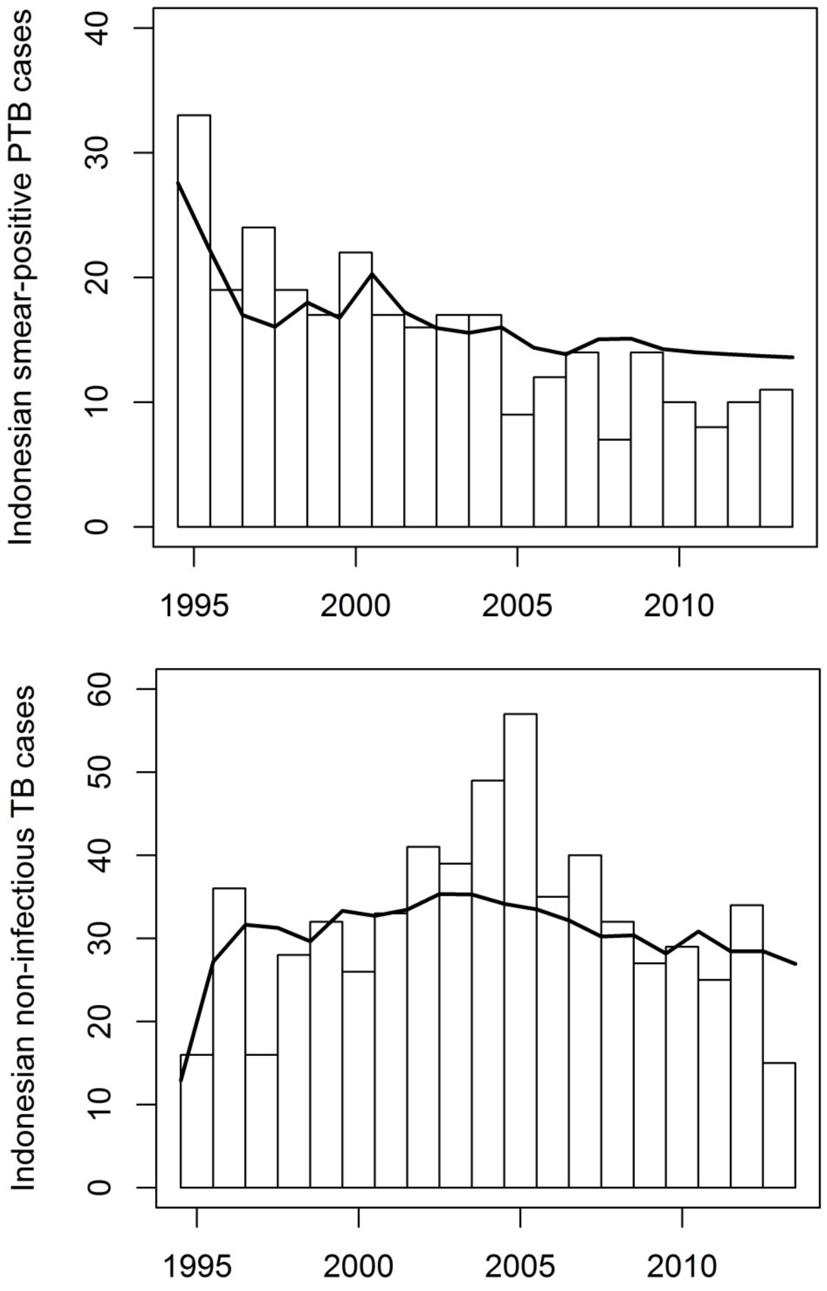
Indonesian smear-positive PTB cases
0
10
20
30
40
1995
2000
2005
2010
Indonesian non-infectious TB cases
0
10
20
30
40
50
60
1995
2000
2005
2010

References

1. Behr MA, Warren SA, Salamon H, Hopewell PC, Ponce de Leon A, Daley CL, et al. Transmission of Mycobacterium tuberculosis from patients smear-negative for acid-fast bacilli. Lancet. 1999;353(9151):444-9.
2. Tostmann A, Kik SV, Kalisvaart NA, Sebek MM, Verver S, Boeree MJ, et al. Tuberculosis transmission by patients with smear-negative pulmonary tuberculosis in a large cohort in the Netherlands. Clin Infect Dis. 2008;47(9):1135-42.
