# Supplementary material for: Latent tuberculosis infection in foreign-born communities: Import vs. transmission in The Netherlands derived through mathematical modelling
Source: PLoS One. 2018 Feb 14;13(2):e0192282. doi: 10.1371/journal.pone.0192282 (PMC5812587; doi:10.1371/journal.pone.0192282)
Supplement: S5 Appendix — (PDF) [file pone.0192282.s009.pdf]

S5 Appendix. Changing diagnosis of EPTB versus PTB and PTB and EPTB over study period: influence of age at diagnosis

Fig A: Average age of TB cases born in Morocco (dots), born in Turkey (red line) and born in Indonesia (blue line).

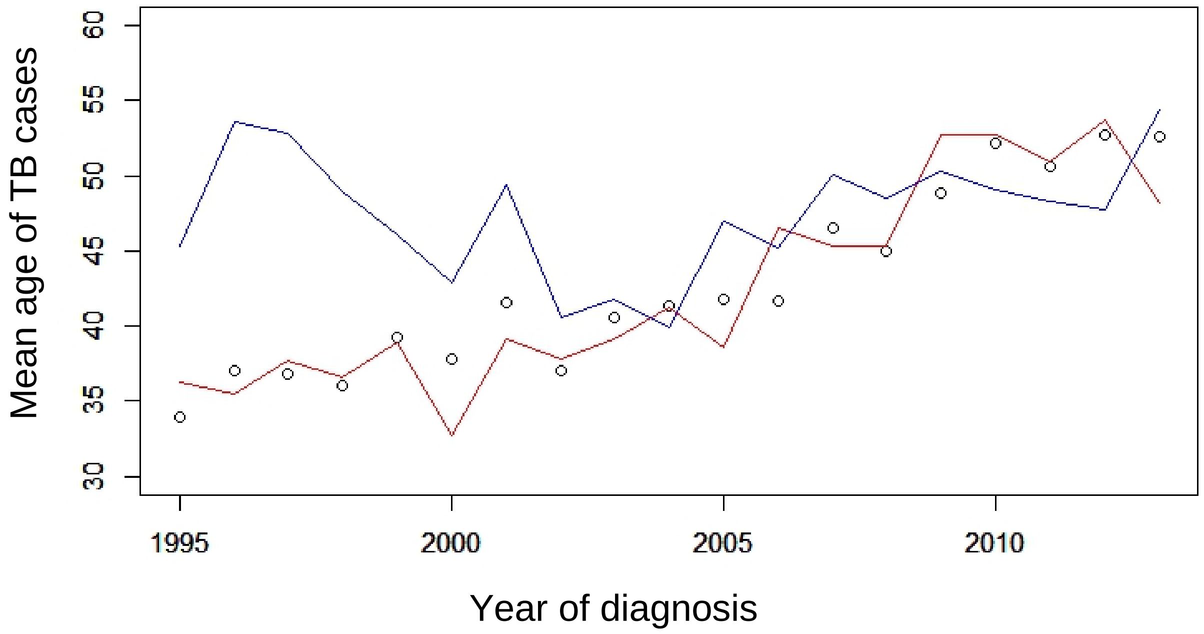

Fig B: Fraction of pulmonary cases (PTB, or PTB and EPTB) versus extrapulmonary cases (EPTB) in Moroccan-born TB cases (A), Turkish-born cases (B) and Indonesian-born (C).

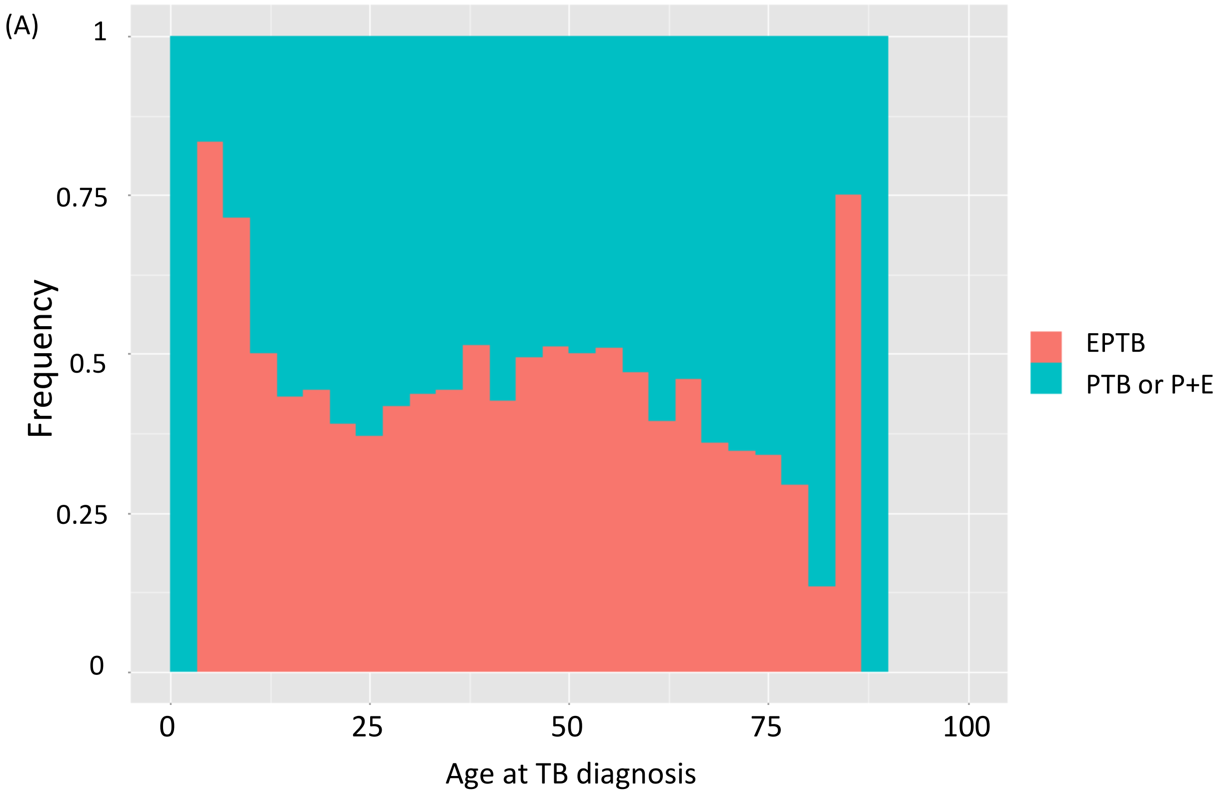

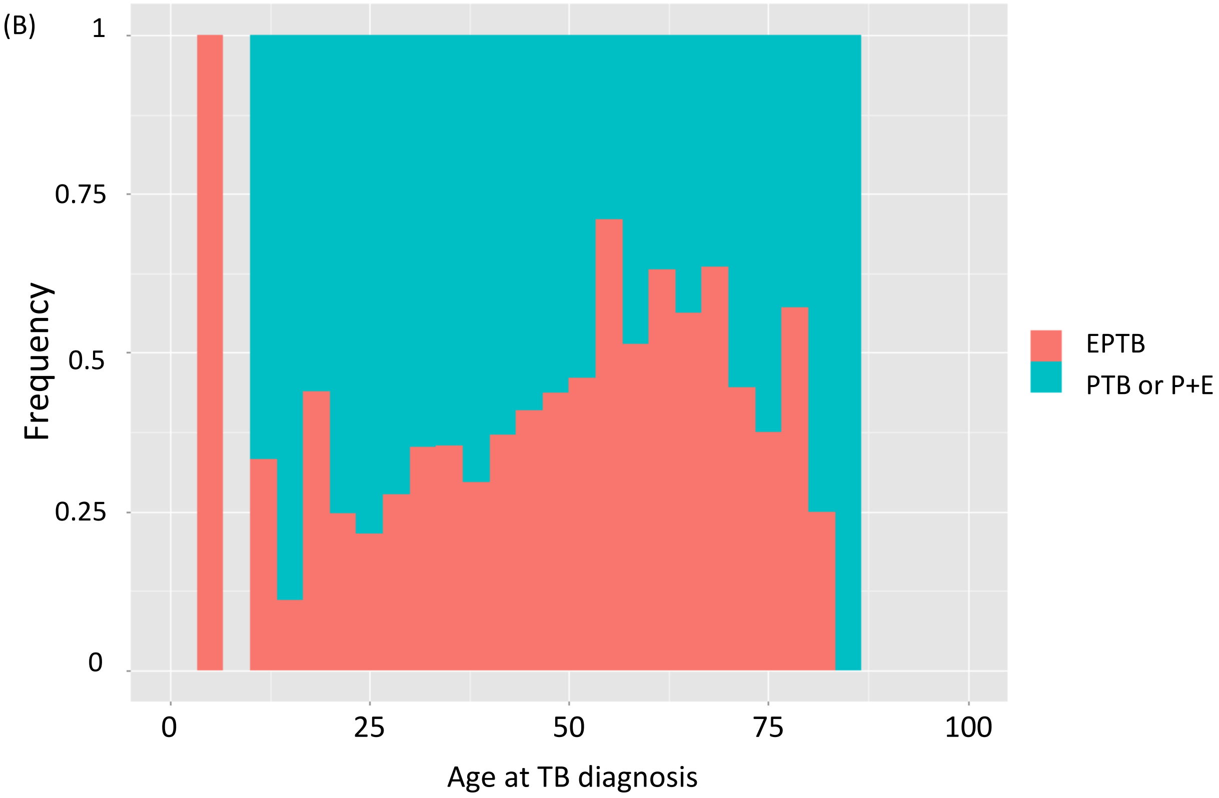

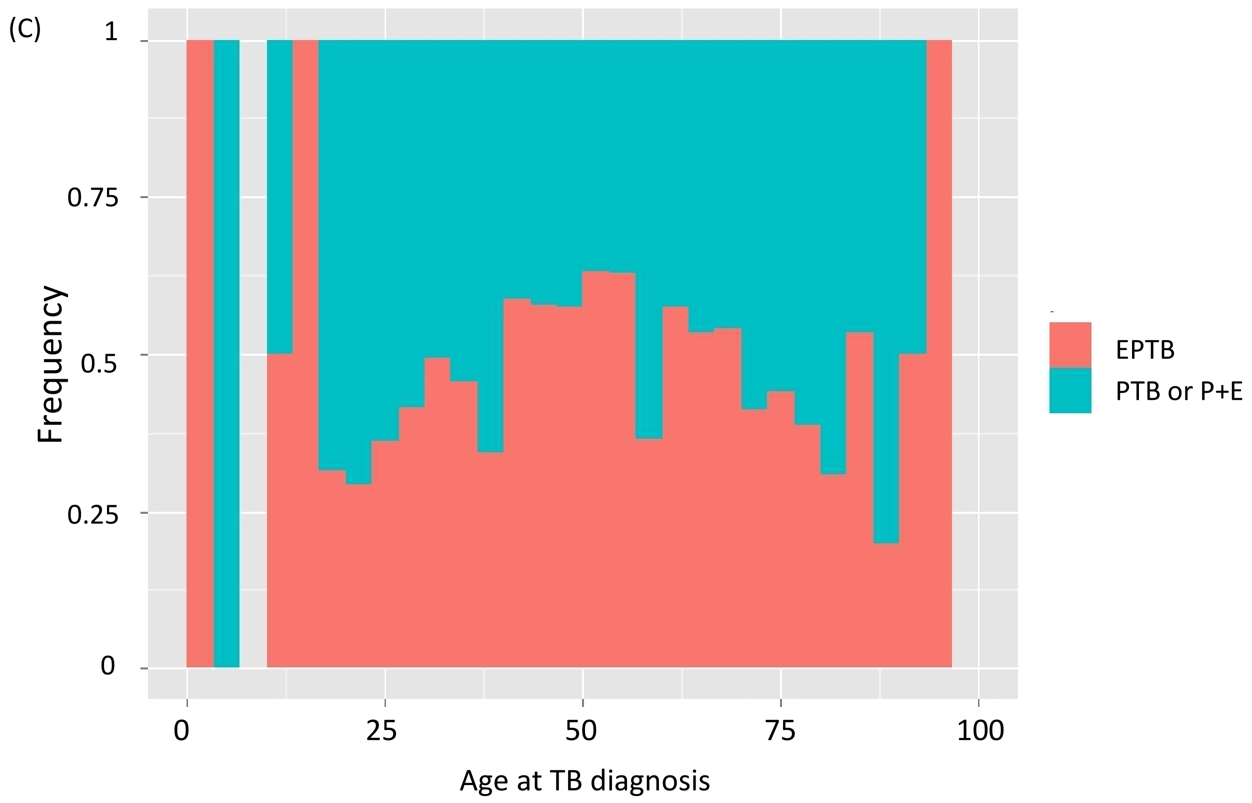
(C)
Frequency
1
0.75
0.5
0.25
0
0
25
50
75
100
Age at TB diagnosis
EPTB
PTB or P+E
