# Supplementary material for: Latent tuberculosis infection in foreign-born communities: Import vs. transmission in The Netherlands derived through mathematical modelling
Source: PLoS One. 2018 Feb 14;13(2):e0192282. doi: 10.1371/journal.pone.0192282 (PMC5812587; doi:10.1371/journal.pone.0192282)
Supplement: S1 Fig — Probability density plots of TB disease by site (pulmonary or extra-pulmonary) and by age, in Moroccan-born TB cases (A), Turkish-born cases (B) and Indonesian-born (C). (PDF) [file pone.0192282.s010.pdf]

S1 Fig: Probability density plots of TB disease by site (pulmonary or extra-pulmonary) and by age, in Moroccan-born TB cases (A), Turkish-born cases (B) and Indonesian-born (C).

(A)

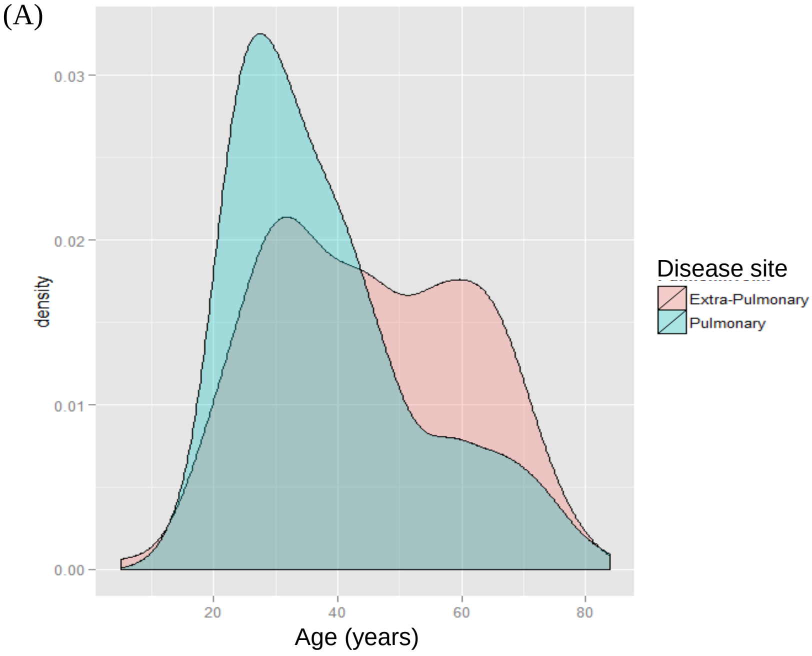

(B)

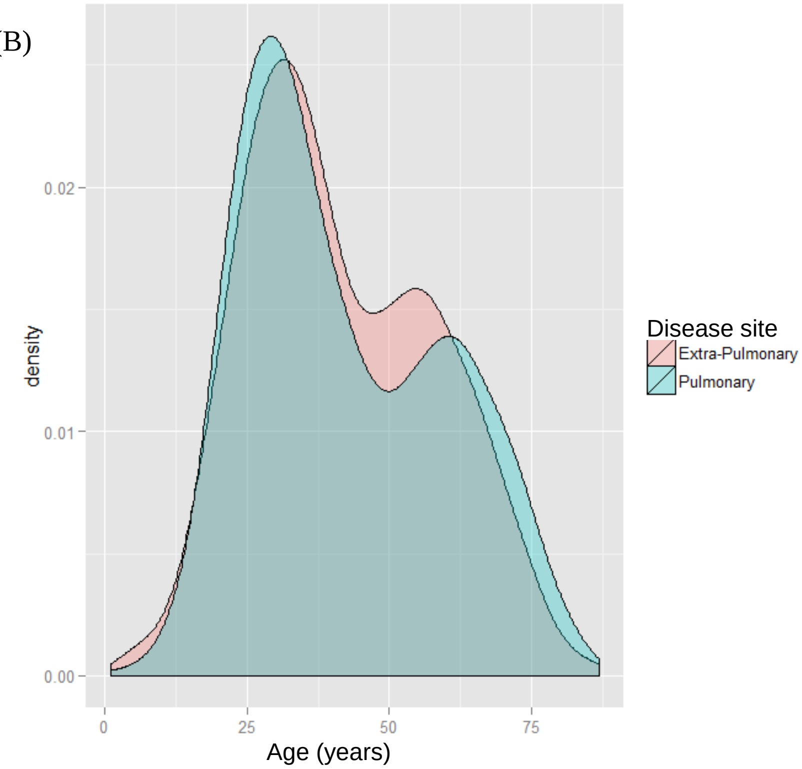

(C)

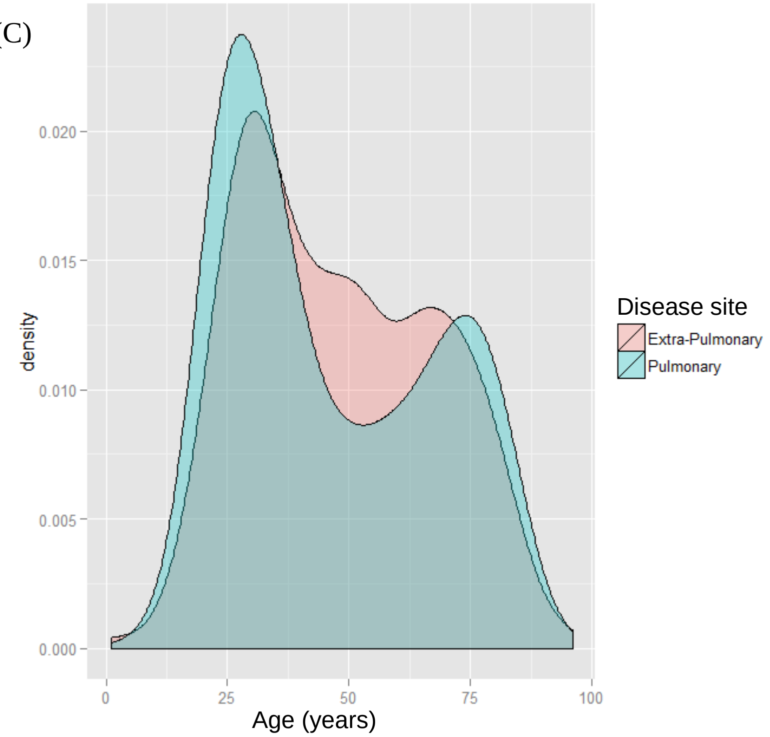
density
0.000
0.005
0.010
0.015
0.020
0
25
50
75
100
Age (years)
Disease site
Extra-Pulmonary
Pulmonary
