# Supplementary material for: Latent tuberculosis infection in foreign-born communities: Import vs. transmission in The Netherlands derived through mathematical modelling
Source: PLoS One. 2018 Feb 14;13(2):e0192282. doi: 10.1371/journal.pone.0192282 (PMC5812587; doi:10.1371/journal.pone.0192282)
Supplement: S2 Fig — Tornadoplot for estimated transmission parameter (1), percentage contribution to LTBI from TB transmission within the Netherlands (2), from immigration (3), or from travel to country of origin (4) for Moroccan (A), Turkish (B) and Indonesians (C). Percentage deviation from the estimate in main text (Table 2) is given for the minimum and maximum values for the parameters in Table 1 in the main text. (PDF) [file pone.0192282.s011.pdf]

S2 Fig: Tornadoplot for estimated transmission parameter (1), percentage contribution to LTBI from TB transmission within the Netherlands (2), from immigration (3), or from travel to country of origin (4) for Moroccan (A), Turkish (B) and Indonesians (C). Percentage deviation from the estimate in main text (Table 2) is given for the minimum and maximum values for the parameters in Table 1 in the main text.

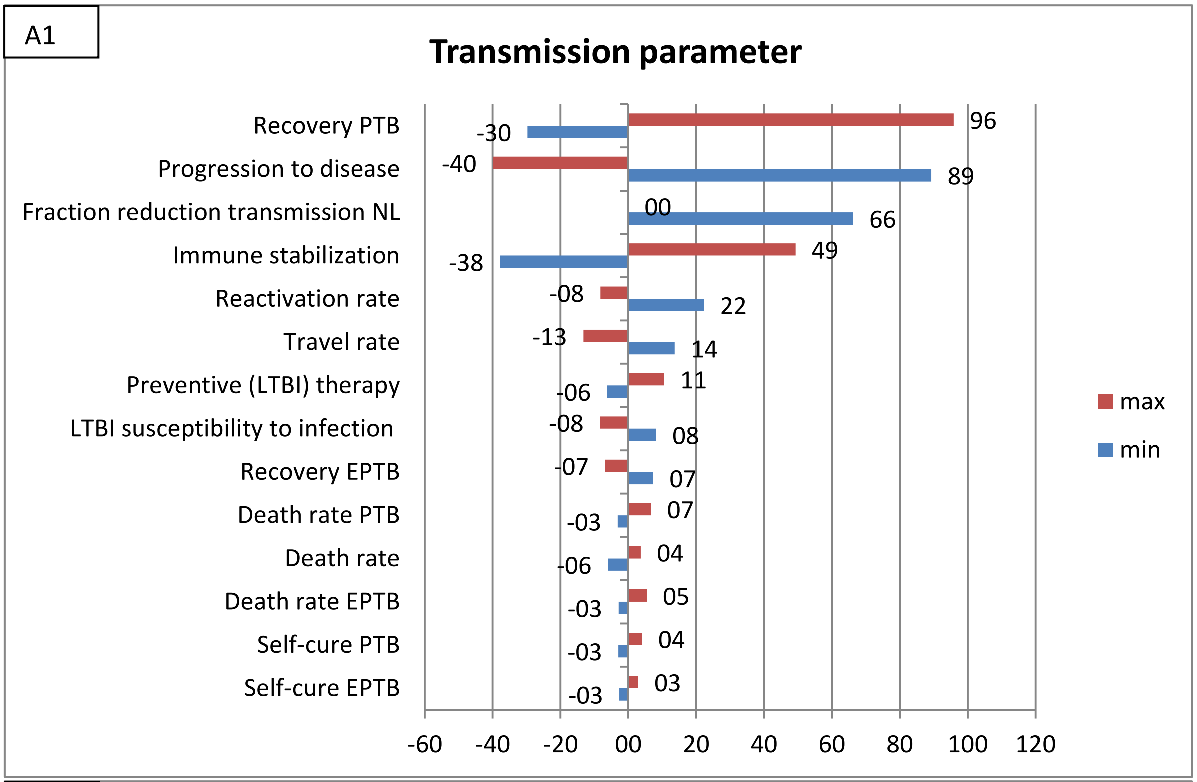

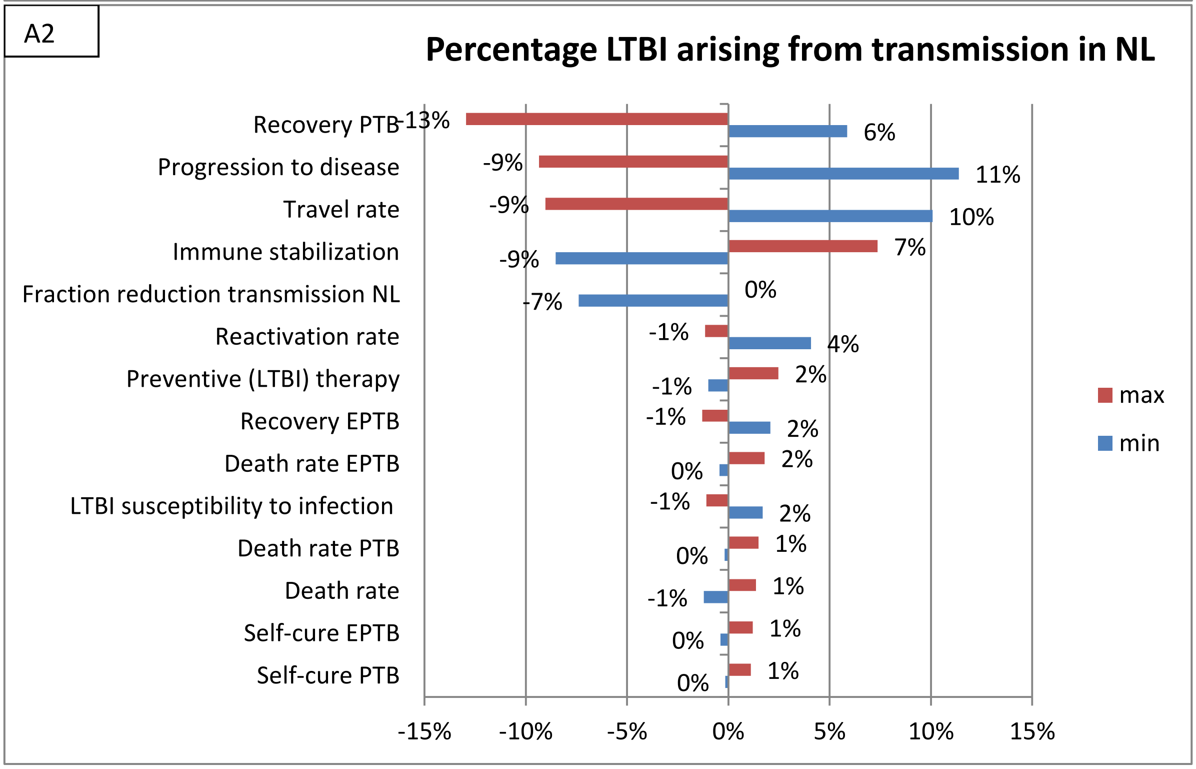

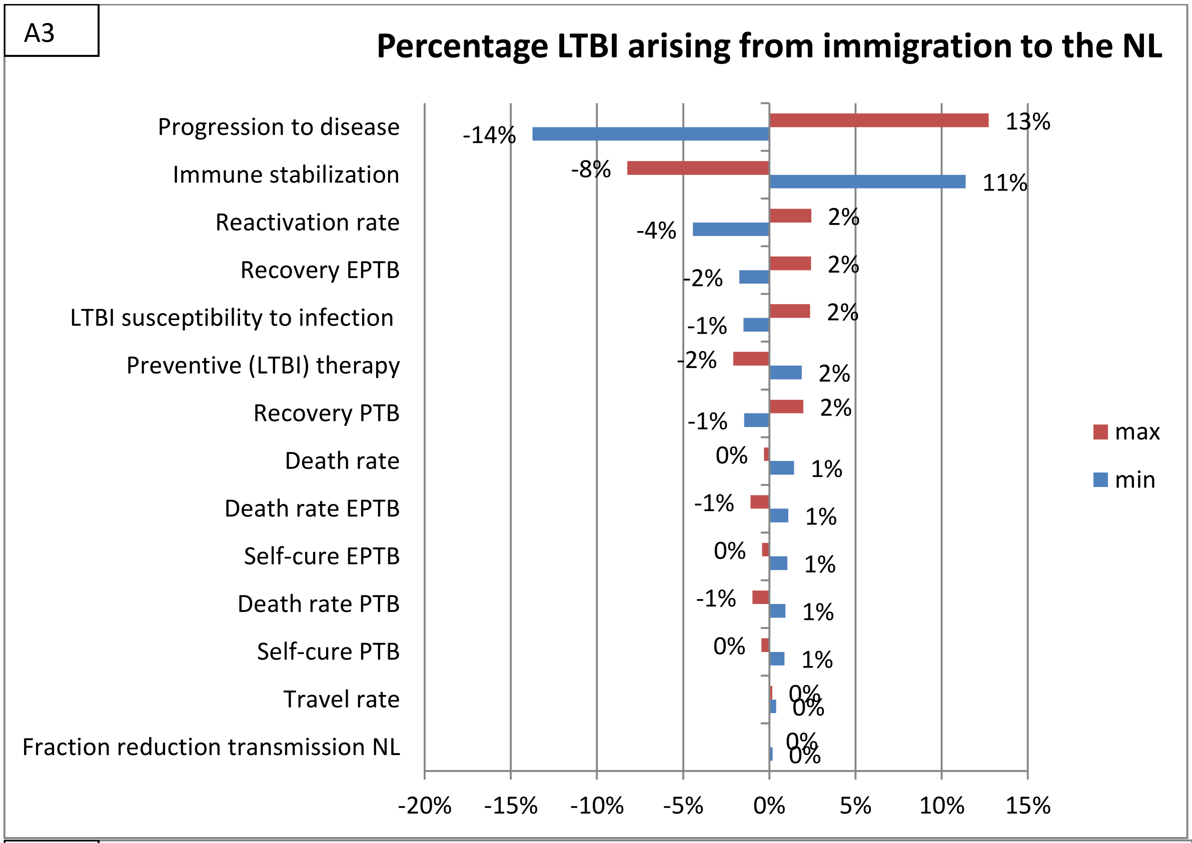
A3
Percentage LTBI arising from immigration to the NL
Progression to disease
Immune stabilization
Reactivation rate
Recovery EPTB
LTBI susceptibility to infection
Preventive (LTBI) therapy
Recovery PTB
Death rate
Death rate EPTB
Self-cure EPTB
Death rate PTB
Self-cure PTB
Travel rate
Fraction reduction transmission NL
-14%
13%
-8%
11%
-4%
2%
-2%
2%
-1%
2%
-2%
2%
-1%
2%
0%
1%
-1%
1%
0%
1%
-1%
1%
0%
1%
0%
0%
0%
0%
-20%
-15%
-10%
-5%
0%
5%
10%
15%
max
min

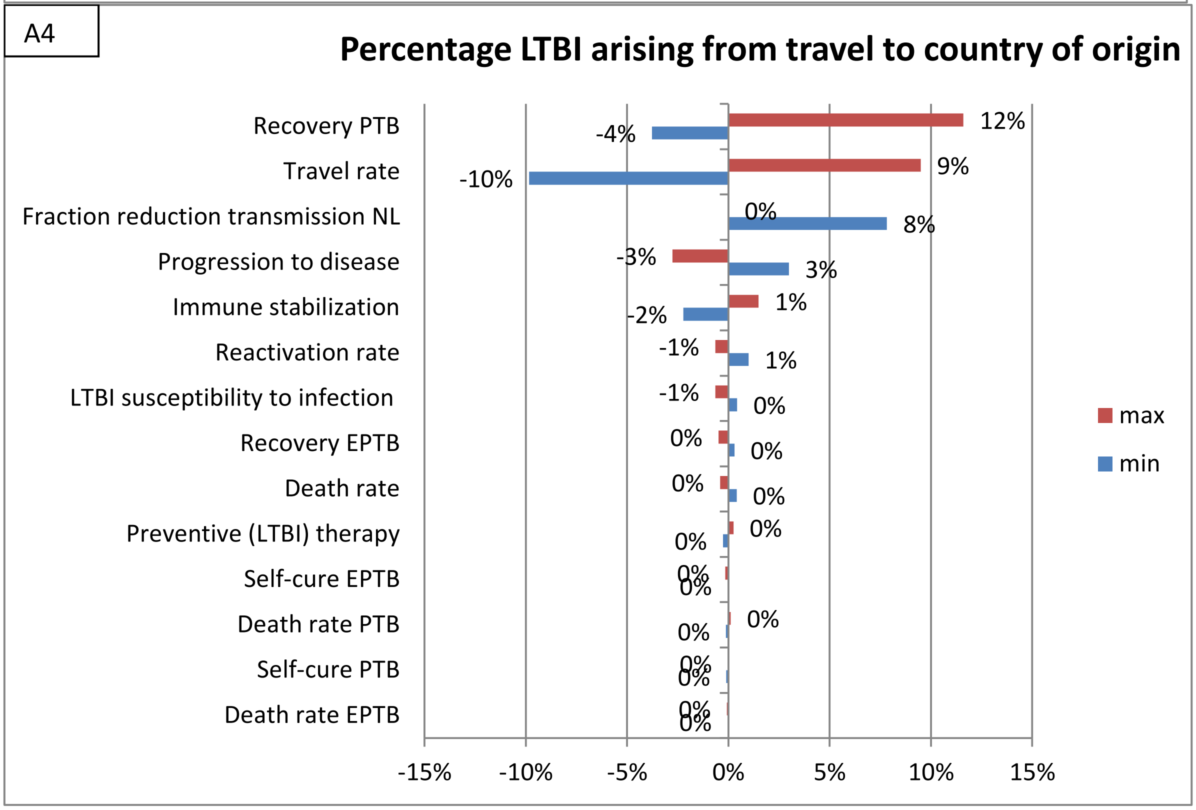
A4
Percentage LTBI arising from travel to country of origin
Recovery PTB
Travel rate
Fraction reduction transmission NL
Progression to disease
Immune stabilization
Reactivation rate
LTBI susceptibility to infection
Recovery EPTB
Death rate
Preventive (LTBI) therapy
Self-cure EPTB
Death rate PTB
Self-cure PTB
Death rate EPTB
-4%
12%
-10%
9%
0%
8%
-3%
3%
-2%
1%
-1%
1%
-1%
0%
0%
0%
0%
0%
0%
0%
0%
0%
0%
0%
0%
0%
0%
0%
-15%
-10%
-5%
0%
5%
10%
15%
max
min

B1

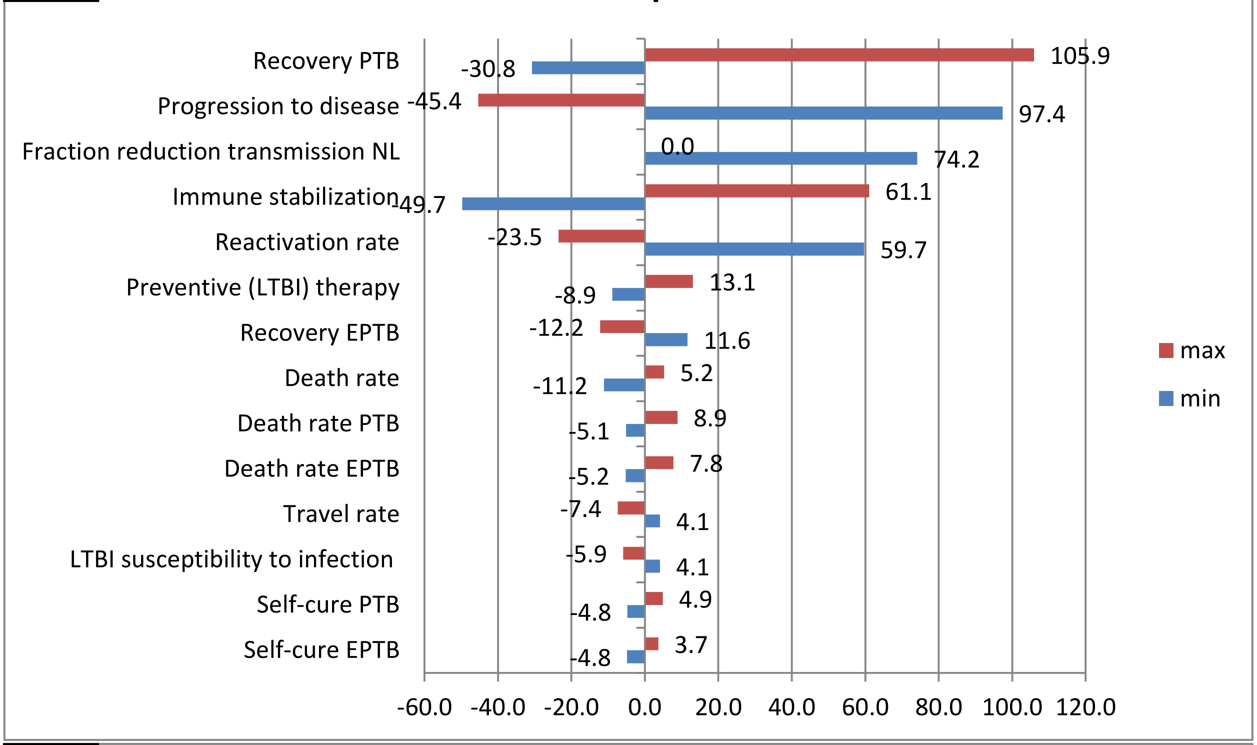
Transmission parameter
Recovery PTB
Progression to disease
Fraction reduction transmission NL
Immune stabilization
Reactivation rate
Preventive (LTBI) therapy
Recovery EPTB
Death rate
Death rate PTB
Death rate EPTB
Travel rate
LTBI susceptibility to infection
Self-cure PTB
Self-cure EPTB
-30.8
105.9
-45.4
97.4
0.0
74.2
-49.7
61.1
-23.5
59.7
-8.9
13.1
-12.2
11.6
-11.2
5.2
-5.1
8.9
-5.2
7.8
-7.4
4.1
-5.9
4.1
-4.8
4.9
-4.8
3.7
-60.0 -40.0 -20.0 0.0 20.0 40.0 60.0 80.0 100.0 120.0
max
min

B2

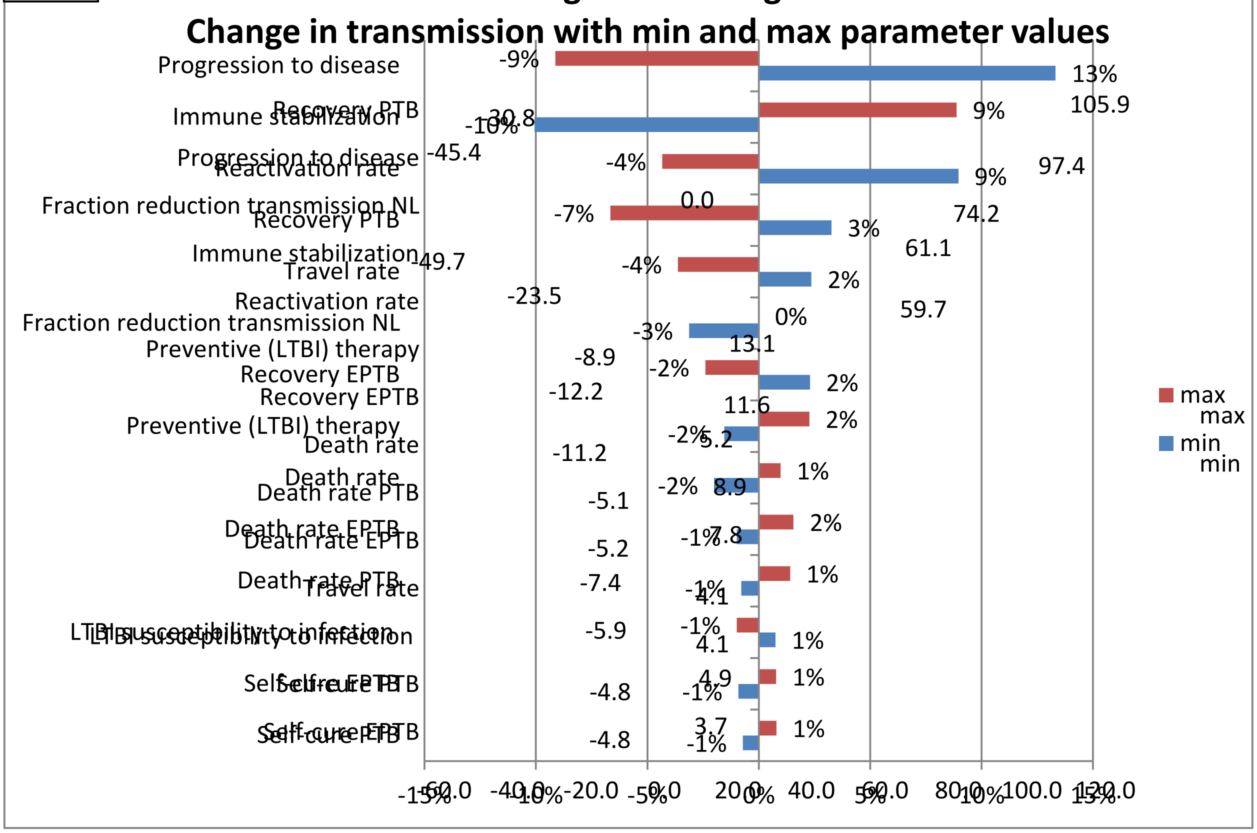
Percentage LTBI arising from transmission in NL
Change in transmission with min and max parameter values
Progression to disease
Immune stabilization
Reactivation rate
Recovery PTB
Travel rate
Fraction reduction transmission NL
Recovery EPTB
Preventive (LTBI) therapy
Death rate
Death rate PTB
Death rate EPTB
LTBI susceptibility to infection
Self-cure EPTB
Self-cure PTB
-9%
13%
9%
-10%
-4%
9%
-7%
3%
-4%
2%
0%
-3%
-2%
2%
2%
-2%
1%
-2%
2%
-1%
1%
-1%
-1%
1%
1%
-1%
1%
-1%
-15% -10% -5% 0% 5% 10% 15%
max
min

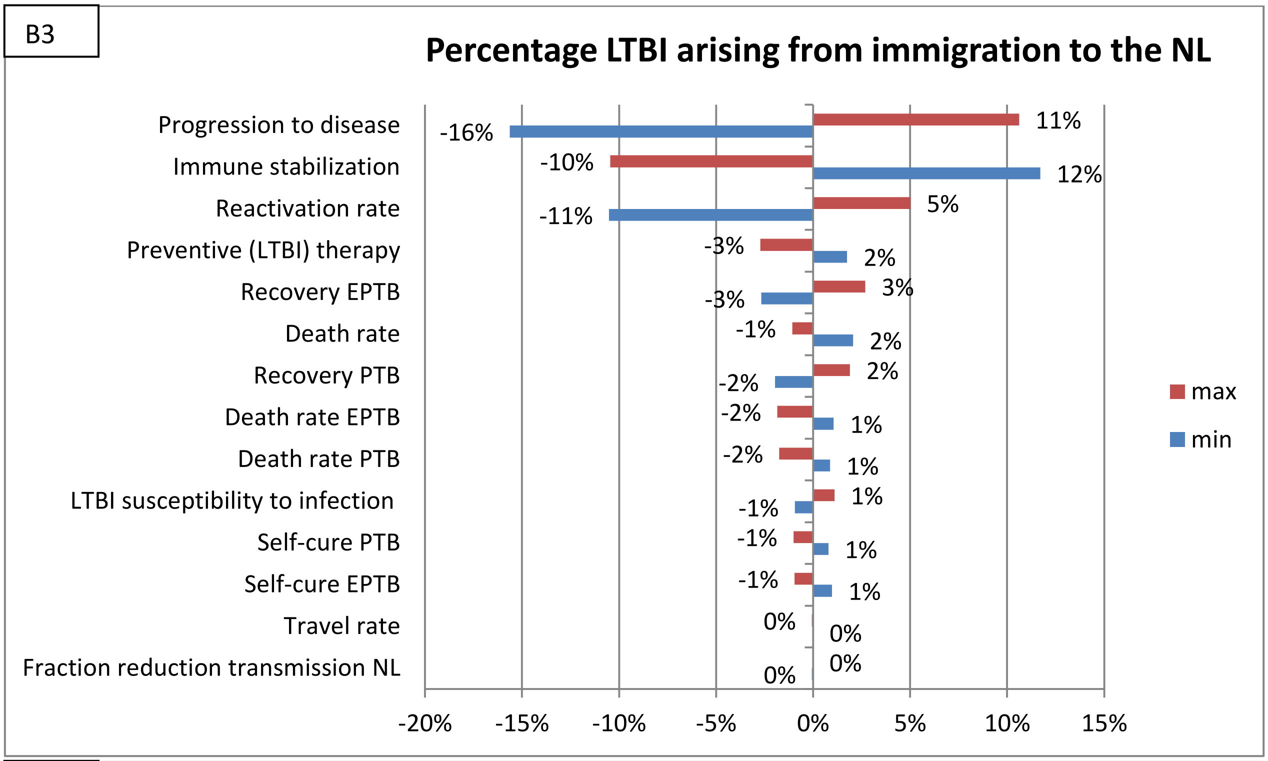
B3
Percentage LTBI arising from immigration to the NL
Progression to disease
Immune stabilization
Reactivation rate
Preventive (LTBI) therapy
Recovery EPTB
Death rate
Recovery PTB
Death rate EPTB
Death rate PTB
LTBI susceptibility to infection
Self-cure PTB
Self-cure EPTB
Travel rate
Fraction reduction transmission NL
-20% -15% -10% -5% 0% 5% 10% 15%
max
min

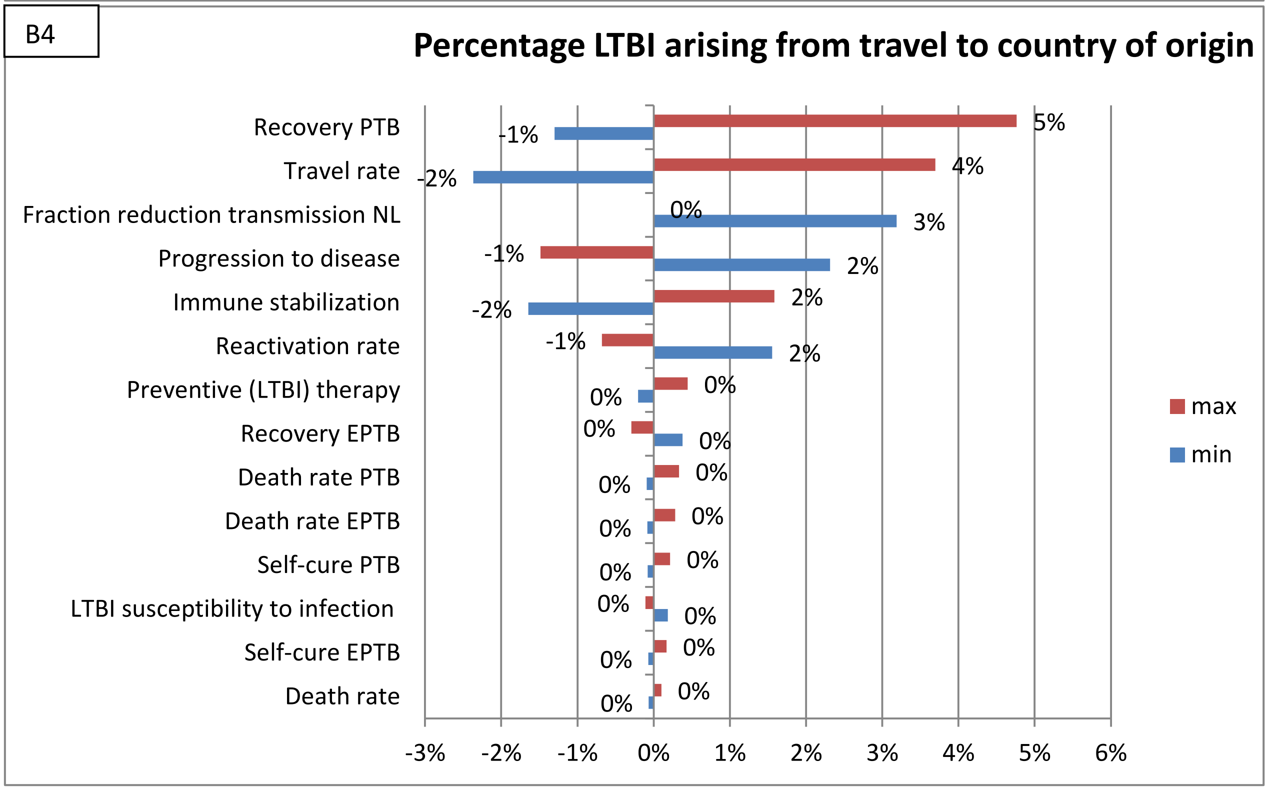
B4
Percentage LTBI arising from travel to country of origin
Recovery PTB
Travel rate
Fraction reduction transmission NL
Progression to disease
Immune stabilization
Reactivation rate
Preventive (LTBI) therapy
Recovery EPTB
Death rate PTB
Death rate EPTB
Self-cure PTB
LTBI susceptibility to infection
Self-cure EPTB
Death rate
-3% -2% -1% 0% 1% 2% 3% 4% 5% 6%
max
min

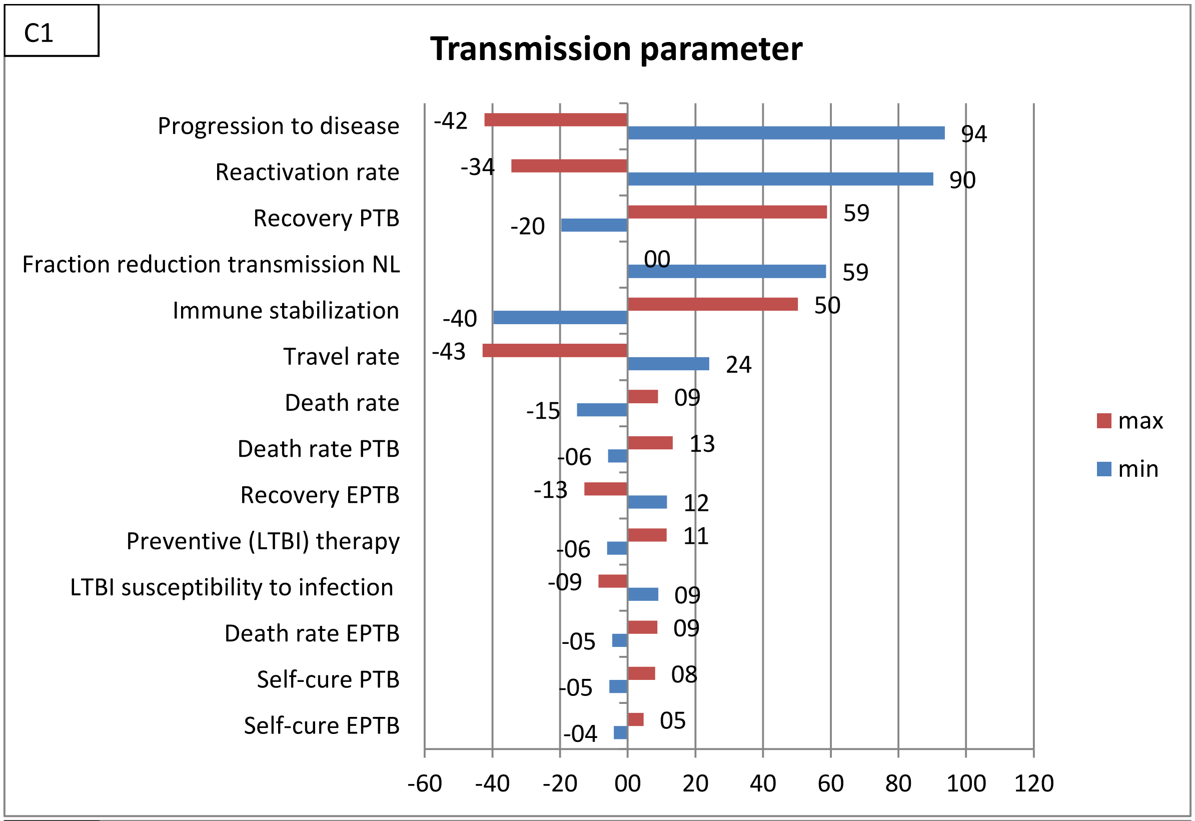
C1
Transmission parameter
Progression to disease
Reactivation rate
Recovery PTB
Fraction reduction transmission NL
Immune stabilization
Travel rate
Death rate
Death rate PTB
Recovery EPTB
Preventive (LTBI) therapy
LTBI susceptibility to infection
Death rate EPTB
Self-cure PTB
Self-cure EPTB
-42
94
-34
90
59
-20
00
59
50
-40
-43
24
09
-15
13
-06
-13
12
11
-06
-09
09
09
-05
08
-05
05
-04
-60
-40
-20
00
20
40
60
80
100
120
max
min

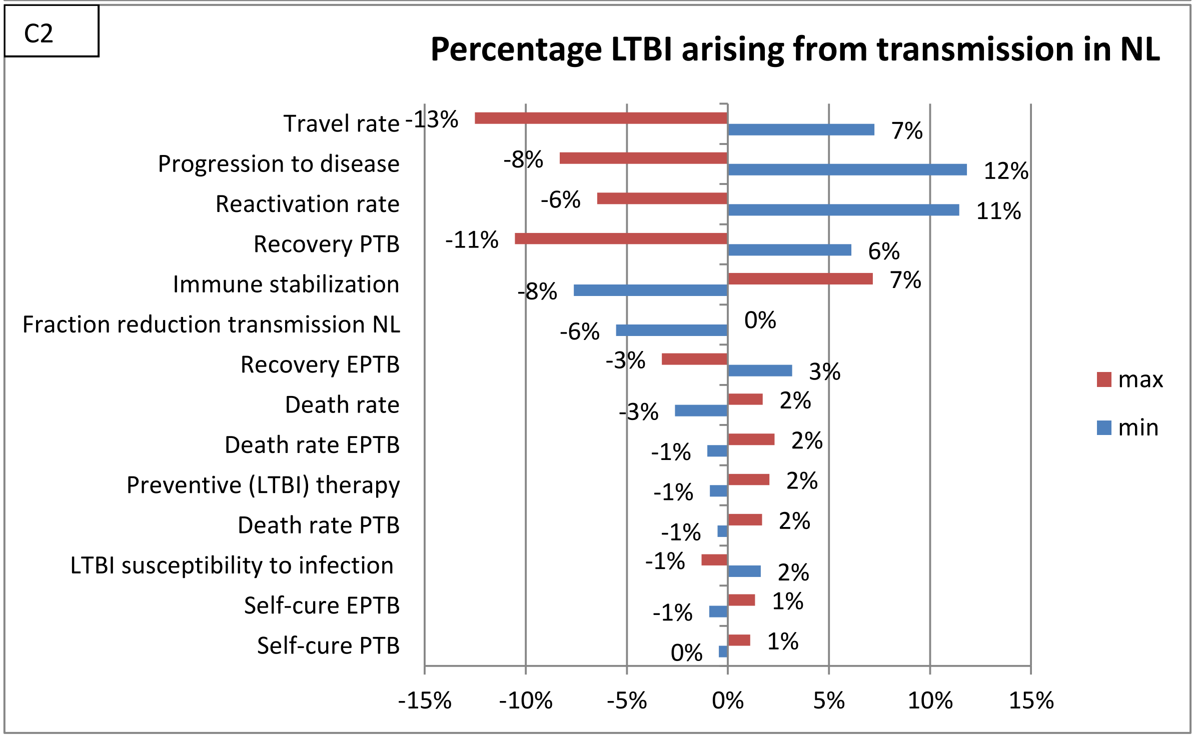
C2
Percentage LTBI arising from transmission in NL
Travel rate
Progression to disease
Reactivation rate
Recovery PTB
Immune stabilization
Fraction reduction transmission NL
Recovery EPTB
Death rate
Death rate EPTB
Preventive (LTBI) therapy
Death rate PTB
LTBI susceptibility to infection
Self-cure EPTB
Self-cure PTB
-13%
7%
-8%
12%
-6%
11%
-11%
6%
7%
-8%
0%
-6%
-3%
3%
2%
-3%
2%
-1%
2%
-1%
2%
-1%
-1%
2%
1%
-1%
1%
0%
-15%
-10%
-5%
0%
5%
10%
15%
max
min
